# Isolated single or pauci-nail lichen planus, three cases

**DOI:** 10.1016/j.jdcr.2024.01.031

**Published:** 2024-02-14

**Authors:** Roy Jiang, Sa Rang Kim, Sean R. Christensen, Amanda Zubek

**Affiliations:** Department of Dermatology, Yale School of Medicine, New Haven, Connecticut

**Keywords:** lichen planus, nail biopsy, nail lichen planus, onychorrhexis

## Introduction

Lichen planus is an inflammatory condition typically limited to skin and oral mucosa; nail involvement occurs in only 1% to 10% of lichen planus cases, and 4% of these cases result in permanent dystrophy.^1^ Multiple nails are usually involved, and 50% of nail lichen planus cases lack accompanying mucocutaneous findings.^2^, ^3^, ^4^, ^5^ Lichen planus involving only a single nail is exceptionally rare, with limited description of this entity in the literature. There is also a paucity of literature discussing how lichen planus involving a single nail should be managed. We sought to present a series of cases of single or pauci-nail lichen planus. We report three cases in which malignancy was initially suspected leading to a biopsy supportive of lichen planus. Patients were treated with intramatrical triamcinolone injections with variable responses in each of the three patients.

## Case 1

**Fig 1:**
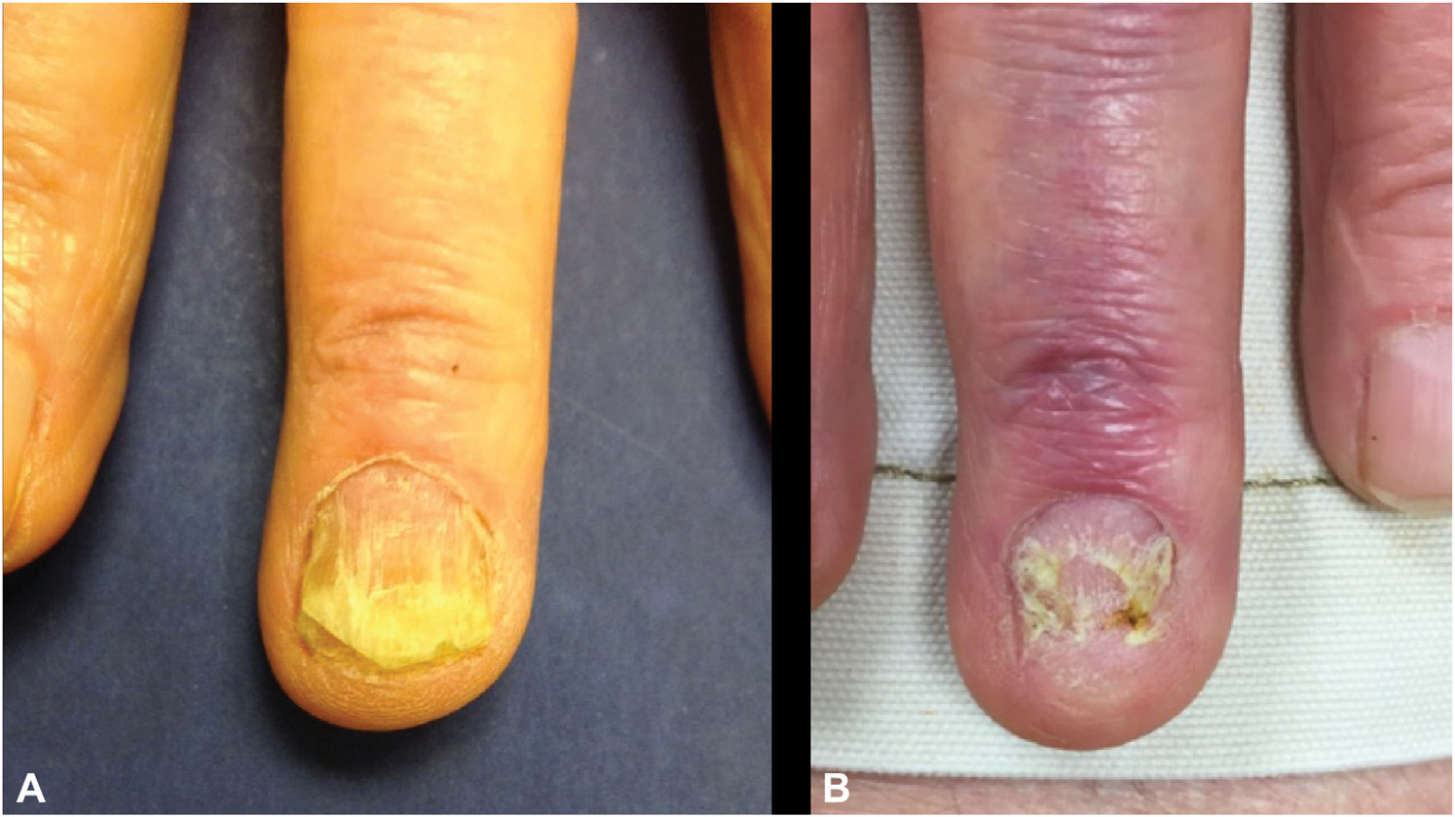
Progressive nail dystrophy of right third (R3) fingernail in case 1. **A,** Examination findings at initial presentation. **B,** Presentation after 1 year demonstrating onycholysis, onychorrhexis, and nail hypoplasia despite intramatrical steroid therapy.

An 83-year-old male presented with right third finger (R3) nail dystrophy. Nail dystrophy had been present for 1 year. The lesion was treated in the past with topical econazole and oral terbinafine without improvement. On exam, R3 fingernail dystrophy was noted (Fig 1, *A*). The nail demonstrated longitudinal and horizontal ridging, splitting, onychoschizia, onycholysis, and subungual debris. The proximal nail fold was pink, mildly edematous, and exhibited loss of eponychial attachment to the nail plate. There was no purulence or bleeding. Given the monodactylous involvement, malignancy was suspected. To assist in diagnosis and to rule out other causes such as irritant-induced paronychia, onychomycosis, or inflammatory nail disease a 4 mm punch biopsy was performed of the distal nail matrix after partial nail avulsion. Histopathology showed band-like lymphocytic infiltrate in the upper dermis supporting a diagnosis of nail lichen planus given the clinical context. Periodic acid-Schiff-stained sections failed to reveal fungi.

The patient was subsequently treated with three injections of intramatrical triamcinolone (between 0.3 and 0.4 mL of 20 to 30 mg/mL triamcinolone) every 6 weeks. The patient continued to present with persistent and stable distal onycholysis, nail yellowing, nail hypoplasia, subungual debris, and longitudinal ridging along the nail (Fig 1, *B*). A periodic acid-Schiff stain of a clipping from the distal nail showed evidence of fungal infection and the patient completed a course of oral terbinafine and topical efinaconazole nail solution. However, given an overall lack of response to antifungal and anti-inflammatory therapy, a nail avulsion was eventually performed to debride the onychomycotic nail plate and provide temporary symptom relief. Despite this procedure, hyperkeratosis, scaling, and hemorrhagic crusting of the nail bed persisted. Further treatment was deferred by the patient as the patient reported that the nail dystrophy at this point was not particularly bothersome.

## Case 2

**Fig 2:**
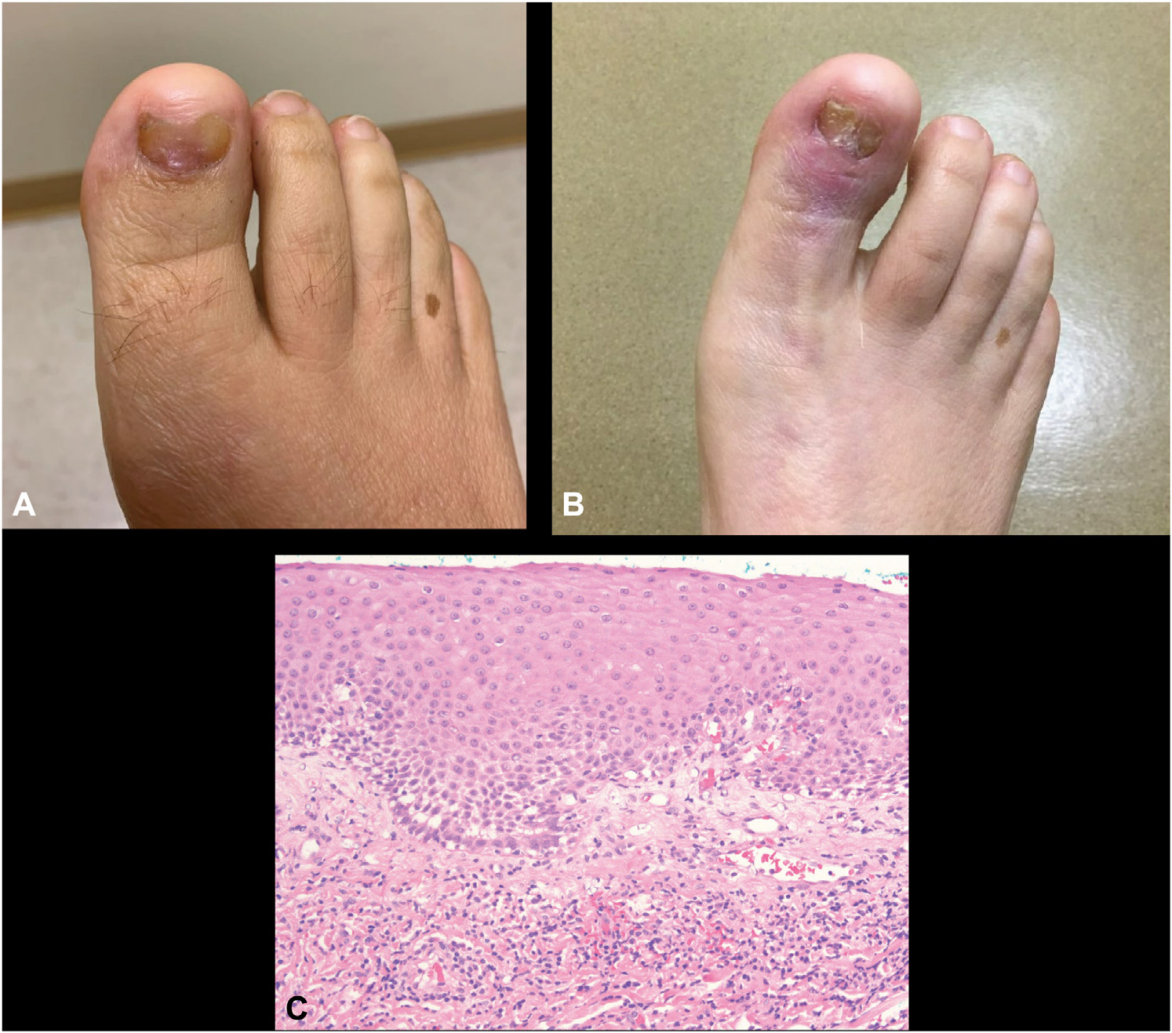
Presentation of right first (R1) toenail nail dystrophy in case 2. **A,** Examination findings 6 months prior to matrix biopsy. **B,** Presentation 2 years after matrix biopsy. **C,** Histopathology of the nail matrix obtained by biopsy of the patient is shown (40×, H&E) with the presence of a band of lymphocytes near the dermal-epidermal junction. *H&E*, Hematoxylin-eosin stain.

A 49-year-old female presented with persistent nail dystrophy of the right first toenail present for 4 months (Fig 2, *A*). Per patient history, the lesion began as a pink subungual papule without pain or discharge. Moderate longitudinal ridging and light tan hyperpigmentation were observed extending up to 3 mm from the proximal nail fold. The remaining 1 cm of the distal nail plate was markedly thinned. The proximal nail fold appeared uninvolved.

The lesion was initially monitored over the course of the next 10 months and eventually biopsied as nail dystrophy worsened. The pink subungual papule was reported to have grown. The lesion eventually involved almost the entire width of the nail with proximal nail plate thinning over the papule. Initial differential diagnosis included trauma, subungual fibroma, and squamous cell carcinoma. After partial nail plate avulsion and reflection of the proximal nailfold, a tangential shave biopsy of the nail matrix was performed. The biopsy demonstrated subepithelial patchy lymphocytic infiltrate supporting a diagnosis of nail lichen planus given clinical findings (Fig 2, *C*).

Healing after the nail biopsy was complicated by increased pain, erythema, and edema without purulence. Bacterial infection was suspected and empirically treated with cephalexin with partial improvement. The nail biopsy site also developed scarring with failure of nail plate formation and development of a dorsal pterygium that was subsequently surgically debrided. Intramatrical triamcinolone injections (5 total injections every 1-3 months, between 0.4 mL-0.7 mL of 10 to 20 mg/mL triamcinolone) were performed along with daily topical clobetasol 0.05% ointment and symptoms of pain and swelling all resolved and the nail plate regrew over the course of 2 years. However, the central portion of the nail plate remained hypoplastic with distal splitting of the nail plate (Fig 2, *B*).

## Case 3

**Fig 3:**
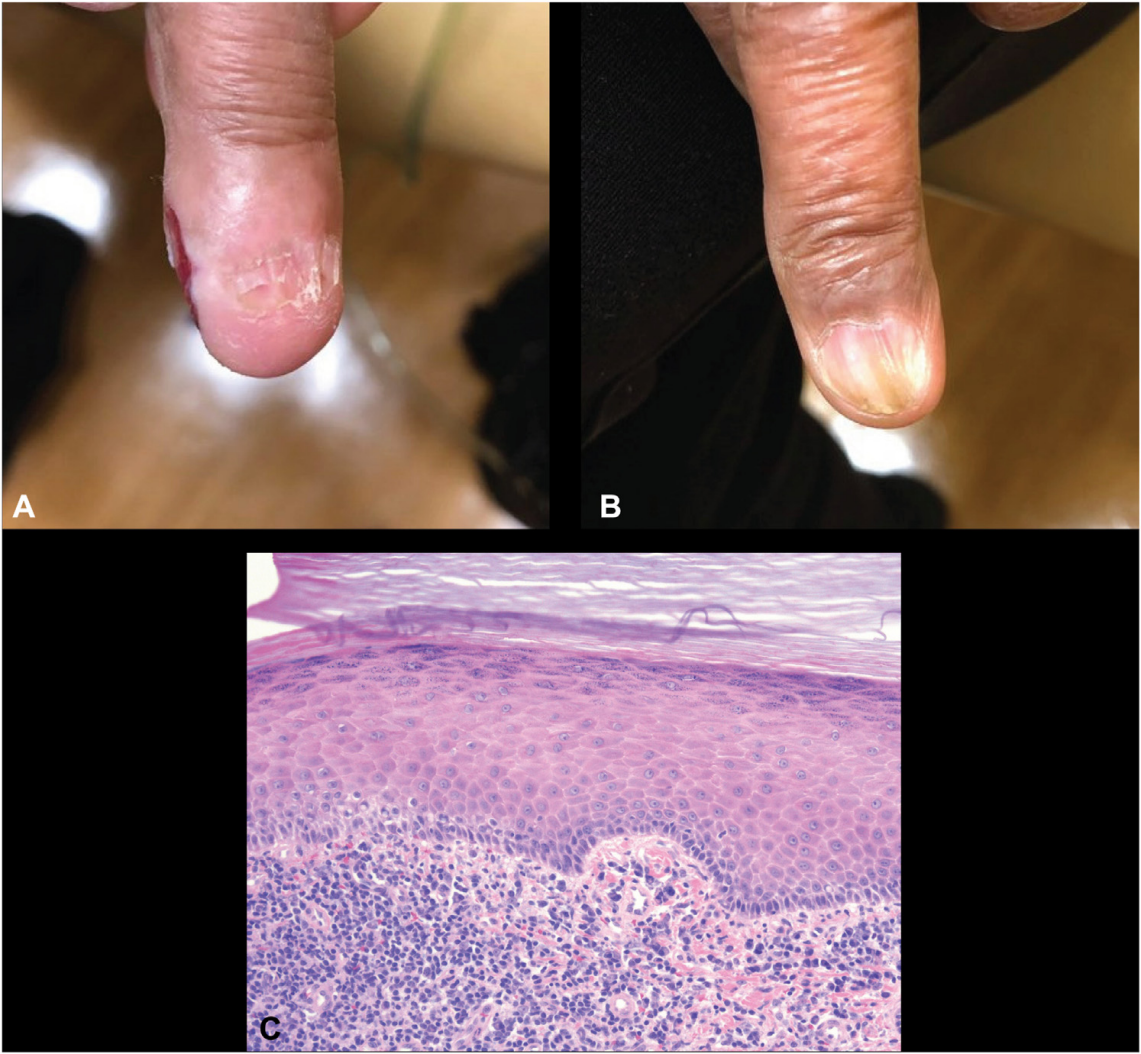
Initial case 3 presentation. **A,** Exam findings of right third (R3) fingernail dystrophy and **B,** ledt third (L3) fingernail dystrophy. Note the simultaneous presence of a herpetic ulcer on the lateral edge of the R3 finger on initial presentation. **C,** Histopathology of the nail eponychium obtained by biopsy of the patient is shown (40×, H&E) with the presence of compact hyperorthohyperkeratosis, focal hypergranulosis, and a band of lymphocytes near the dermal-epidermal junction. *H&E*, Hematoxylin-eosin stain.

A 66-year-old female with a history of chronic interstitial lung disease managed with mycophenolate and low dose oral prednisone presented with a 6-month history of nail dystrophy involving both the right third and left third fingernails. On examination, the R3 fingernail appeared to be obliterated with only residual hypoplastic nail (Fig 3, *A*). The left third fingernail displayed longitudinal ridging with hypoplasia and a central 2 mm brown longitudinal streak (Fig 3, *B*). Physiologic melanonychia was also observed on multiple normal appearing nails. Of note, the patient also presented with a chronic Herpes simplex virus (HSV) infection resulting in ulceration of the R3 lateral finger that resolved following 6 months of famciclovir treatment (Fig 3, *A*).

A 4 mm punch biopsy of the distal nail matrix of the R3 finger was performed through the proximal nailfold, nail plate, and underlying distal matrix given a concern for possible malignancy or dystrophy secondary to HSV infection. The biopsy demonstrated a lichenoid infiltrate of lymphocytes obscuring the dermal-epidermal junction adjacent to the matrix, along with epidermal hyperplasia, focal hypergranulosis, and compact hyperorthokeratosis suggestive of lichen planus (Fig 3, *C*). The microscopic differential diagnosis included lichenoid changes adjacent to herpes virus infection. However, viral cytopathic changes were absent on matrix biopsy. Given the suspected diagnosis of nail lichen planus, the patient underwent 2 rounds of intramatrical triamcinolone (0.25 mL of 5 mg/mL triamcinolone) for both nails every 8 weeks but was subsequently lost to follow-up.

## Discussion

Early identification of single nail lichen planus and treatment to prevent significant morbidity are important goals for improving the management of this condition. We present here 2 cases of isolated nail dystrophy involving a single nail as well as a case of nail dystrophy affecting a single nail on each hand. While malignancy was suspected or considered in all 3 cases, this series suggests that inflammation of the nail unit suggestive of lichen planus can be the cause of monodactylous or single nail changes.

Nail dystrophy involving a single nail should raise clinical suspicion for the possibility of malignancy such as squamous cell carcinoma or amelanotic melanoma of the nail unit in addition to inflammatory or infectious causes.^6^^,^^7^ Nail unit biopsies may carry a higher risk of morbidity than cutaneous biopsies, and reluctance to perform a biopsy for this reason can delay treatment. Given the possibility of malignancy causing monodactylous onychodystrophy, a biopsy for single nail dystrophy should be pursued.

Lichen planus isolated to the nails typically involves multiple digits. Three large cohort studies involving 20, 21, and 24 patients (respectively) with nail lichen planus did not report any cases limited to single nails.^2^^,^^3^^,^^5^ A study by Tosti et al of 24 patients with isolated nail lichen planus identified no patients with single nail involvement.^3^ Two other studies have alluded to the possibility of single nail involvement in lichen planus. A study by Goettmann et al involving 67 patients with nail lichen planus reported that 8 patients experienced involvement of a single fingernail. However, no cases of involvement of a single toenail were reported, and these 8 patients were not examined further.^4^ Biopsies were performed in all 67 cases in the study by Goettmann et al^3^, ^4^ In a study by Piraccini et al, 1 case out of a total of 75 cases was documented as involving only a single fingernail (the patient benefitted from intralesional steroid injection).^8^

As in our case series, longitudinal ridging and nail thinning were the most common findings (90%) associated with nail lichen planus across multiple studies.^3^, ^4^ Hyperkeratosis and onycholysis may also be seen—albeit at a lower frequency (around 40%).^3^, ^4^ The prognosis of nail lichen planus is marked by frequent persistence or progression of nail disease as in the first case presented here.^4^^,^^9^ In the study by Tosti et al, spontaneous resolution occurred in only 1 out of 20 patients; relapses occurred in 8 patients of 15 treated with oral/intramuscular triamcinolone and 4 patients with intralesional injection at 2 years.^3^ Piraccini et al demonstrated that in a cohort of 75 patients, two-thirds of patients responded to treatment with systemic steroids but 60% of patients relapsed.^8^ In Goettmann et al, only 13 patients completely recovered and 29 patients improved out of a total of 67. Most patients were treated systemically (69%) and local treatment was reserved for patients with involvement limited to only a few nails.^4^ Nevertheless, 70% of these patients relapsed, mostly within the first year.

Other systemic treatments that have been investigated for nail lichen planus include antimalarials,^10^ oral retinoids,^11^^,^^12^ cyclosporine,^13^ and etanercept^14^ but these treatments are often accompanied by relapse. Current recommendations regarding treatment depend on the extent of nail involvement^15^ with systemic therapy reserved for patients with more than three nails involved. First line treatments are intralesional triamcinolone for cases with only matrix involvement, but intramuscular corticosteroid injection can be considered if the nail bed is also involved or if multiple nails are affected; second line treatments include oral retinoids.^9^

In conclusion, a diagnosis of isolated nail lichen planus should be considered in patients with persistent dystrophic single nail involvement on 1 or both hands. The simultaneous presence of onychomycosis or HSV should not rule out a diagnosis. Current management is challenging with high rates of relapse and persistent nail dystrophy even when only a single nail is involved. Overall, the rarity of single or pauci-nail lichen planus makes diagnosis challenging due to availability bias. Early identification via biopsy of single nail lesions prior to progression or complete nail destruction as seen in this series, may be critical to improving outcomes.^9^^,^^15^

## Conflicts of interest

None disclosed.

## References

[1] Zaias N. (1970). The nail in lichen planus. Arch Dermatol.

[2] Chiheb S., Haim H., Ouakkadi A., Benchikhi H. (2015). Caractéristiques cliniques et évolutives du lichen plan unguéal: étude descriptive de 20 patients. Ann Dermatol Venereol.

[3] Żychowska M., Żychowska M. (2021). Nail changes in lichen planus: a single-center study. J Cutan Med Surg.

[4] Goettmann S., Zaraa I., Moulonguet I. (2012). Nail lichen planus: epidemiological, clinical, pathological, therapeutic and prognosis study of 67 cases. J Eur Acad Dermatol Venereol.

[5] Tosti A., Peluso A.M., Fanti P.A., Piraccini B.M. (1993). Nail lichen planus: clinical and pathologic study of twenty-four patients. J Am Acad Dermatol.

[6] André J., Moulonguet I., Goettmann-Bonvallot S. (2010). In situ amelanotic melanoma of the nail unit mimicking lichen planus. Arch Dermatol.

[7] Levandoski K.A., Nazarian R.M., Asgari M.M. (2017). Hypertrophic lichen planus mimicking squamous cell carcinoma: the importance of clinicopathologic correlation. JAAD Case Rep.

[8] Piraccini B.M., Saccani E., Starace M., Balestri R., Tosti A. (2010). Nail lichen planus: response to treatment and long term follow-up. Eur J Dermatol.

[9] Iorizzo M., Tosti A., Starace M. (2020). Isolated nail lichen planus: an expert consensus on treatment of the classical form. J Am Acad Dermatol.

[10] Mostafa W.Z. (1989). Lichen planus of the nail: treatment with antimalarials. J Am Acad Dermatol.

[11] Alsenaid A., Eder I., Ruzicka T., Braun-Falco M., Wolf R. (2014). Successful treatment of nail lichen planus with alitretinoin: report of 2 cases and review of the literature. Dermatology.

[12] Iorizzo M. (2016). Nail lichen planus - a possible new indication for oral alitretinoin. J Eur Acad Dermatol Venereol.

[13] Florian B., Angelika J., Ernst S.R. (2014). Successful treatment of palmoplantar nail lichen planus with cyclosporine. J Dtsch Dermatol Ges.

[14] Irla N., Schneiter T., Haneke E., Yawalkar N. (2010). Nail lichen planus: successful treatment with etanercept. Case Rep Dermatol.

[15] Lipner S.R. (2019). Nail lichen planus: a true nail emergency. J Am Acad Dermatol.

